# How to make an eye unit child friendly

**Published:** 2010-03

**Authors:** Joan McLeod-Omawale, Alamgir Hossain

**Affiliations:** Senior Technical Advisor/LAC Director, ORBIS International, 520 8th Ave, 11th Floor, New York, NY 10018, USA.; Programme Coordinator, BCCC, Sightsavers International, Apt 5B and 5C, House 7, Road 33, Gulshan, Dhaka 1212, Bangladesh.

**Figure:**
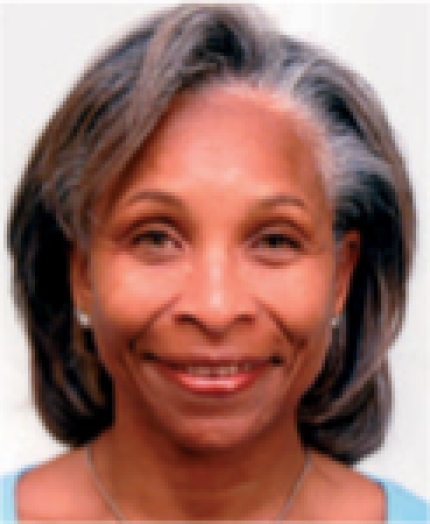


**Figure:**
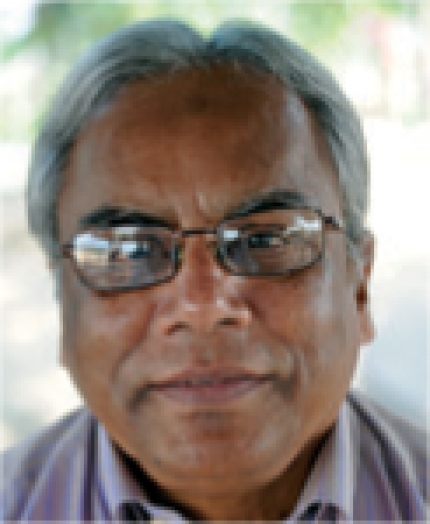


Children are not simply small adults - they have very different needs! Children are more easily frightened than adults, they get restless and irritable more quickly than adults, and they have a need to play and explore their environment.

Parents and carers also need support in the eye care environment. Mothers of young babies need to have space and privacy for breastfeeding, for example. Parents also need to know what is happening to their child and what is required of them.

Meeting the needs of children and their parents in an eye unit, whether at primary, district, or tertiary level, has a significant impact on the eye team's ability to provide good quality eye care.

At the most basic level, a friendly atmosphere and thoughtful treatment of children and parents will:

- Reduce fear of hospitals and doctors among both children and parents, which encourages parents to bring their children back for necessary and important referral or follow-up visits.
- Reduce children's distress, which will allow doctors to examine them better.

The authors have been involved in setting up sophisticated child eye units in India and Bangladesh. These eye units are designed to meet the specific needs of children but are very expensive, costing between US $15,000 and US $20,000 each. In this article, the authors hope to share the key principles of making an eye unit child friendly. Even on a very small budget, there is much that can be done to improve the eye care experiences of children.

## Space to play

Children should be able to spend waiting time doing enjoyable things such as playing with toys, looking at pictures, or reading stories (some of which may include a health education component). Children who enjoy their visit will be more willing to come back for follow-up!

You can produce your own simple toys, such as dolls made from cloth (beware of buttons as they can be a choking hazard), or wooden blocks painted with non-toxic paints. Ask for donations of books and toys from local well-wishers, the community, or places of worship. Provide a play area that is safe from sharp corners and has soft, comfortable flooring where children can sit down and play (Figure 1). This needn't be expensive - even a blanket will be better than a hard, cold floor!

**Figure:**
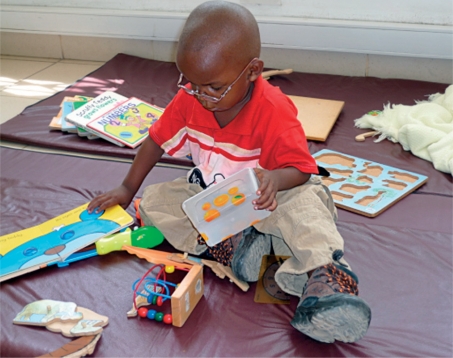
Figure 1. Provide a play area that is safe and comfortable

Clare Gilbert

Encourage local artists to draw colourful pictures, cartoons, or slogans on the walls. All images and text should be appropriate for children and sensitive to the local culture, perhaps using popular fictional or real-world figures. Figure 2 shows an example from Africa and Figure 3 one from India.

**Figure:**
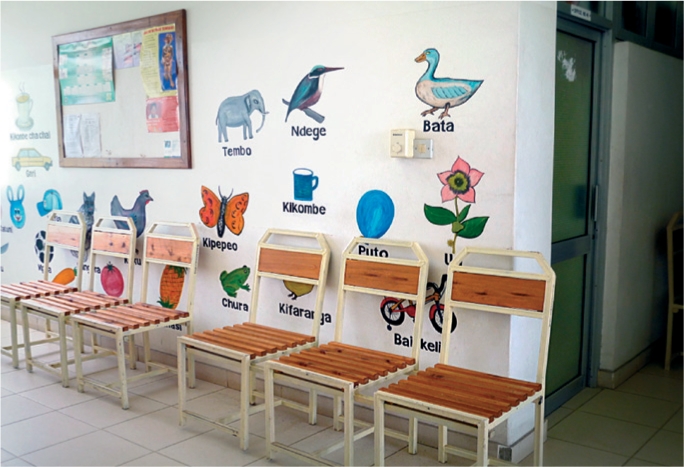
Figure 2. Drawings of animals and objects, Tanzania.

Clare Gilbert

**Figure:**
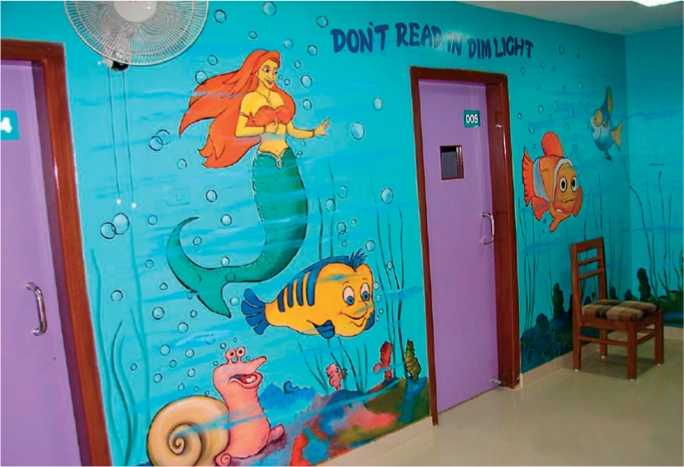
Figure 3. Popular cartoon figures in a child eye unit in Assam, India

Sri Sankaradeva Nethralaya, Assam

## Shorter waiting times

A child-friendly eye clinic should be sensitive to the need for timely care. Long waiting times may contribute to children's boredom and/or distress.

Ideally, services for children should all be provided in one place. For example, registration of children can be in the children's unit rather than in the main registration area. Medical records can also be kept in the children's unit and children's spectacles could be available on site.

In a general eye unit, make time to see children first! Provide a separate queuing area for mothers and children and ensure that people know that children will be seen first - this may encourage parents to bring their children.

It is helpful to test children's visual acuity (VA) in a separate area, away from adults, as it is less distracting for them. Testing VA in children is also more time-consuming. If possible, one member of the team should be trained in testing VA in children and assigned to do this.

## Child-friendly facilities

Ideally, the eye unit should be safe, clean, spacious, colourful, attractive and enjoyable, with child-sized furniture and bathrooms. To achieve this on a small budget:

- Paint different-sized crates or wooden boxes in bright, contrasting colours (using lead-free paint) or cover them in strong fabric to create child-sized tables and seats. Ensure that all sharp edges and nails are removed first!
- Provide a small step or sturdy box (approximately 15–20 cm high) to help children use an adult toilet and wash basin. The box can also help children ‘reach’ the slit lamp. Remind parents that these should be used under the supervision of an adult.

## Parent-friendly facilities

Depending on the cultural context, there could be a separate, quiet room with comfortable seating for breastfeeding mothers. A low-cost alternative is to hang a curtain across a corner or section of the waiting room to create a private space.

Parents will also appreciate a sturdy table or enough floor space for changing nappies - this can be in the bathroom nearest the waiting area. Provide a basin for hand washing.

## Equipment and technology

The outpatient department and the operating theatre should be fully equipped so that children can be adequately examined and assessed, and undergo high-quality surgery.

The IAPB Standard List, 2009 edition, has separate sections for the equipment and consumables needed in a child eye care centre (see Useful Resources on page 11).

The examination room(s) should have a table or patient chair that can be raised and lowered as required and can also be used for supine examination of infants.

Consumables appropriate for children should be available, such as paediatric spectacle frames and small-diameter, high-power intraocular lenses. Many of these can be purchased through the ICEE Global Resource Centre in Durban, South Africa (see Useful Resources on page 11).

## Child- and parent-friendly staff

Identify, support, and reward staff who are good at dealing with children. Train all staff to be welcoming, caring, and supportive of children and their parents.

Encourage all staff to wear casual clothes instead of uniforms - preferably no hats or caps!

Encourage all staff to communicate with children and their parents. Children will respond if you are friendly, even if they can't understand what you say. If you are friendly with the parents, this will help to win the child's trust.

Most parents will need your help to understand what they have to do, whether it is to instil eye drops regularly, to take their child to a referral centre, or to bring the child back for follow-up.

It may be helpful to have written information available which explains the more common eye conditions of childhood. However, some parents may struggle to read for various reasons - it is never a good idea to rely on such materials alone. You still need to talk to the parents or carers yourself; the materials are merely there to support you and reinforce your message.

